# Equal pain—Unequal fear response: enhanced susceptibility of tooth pain to fear conditioning

**DOI:** 10.3389/fnhum.2014.00526

**Published:** 2014-07-18

**Authors:** Michael L. Meier, Nuno M. P. de Matos, Mike Brügger, Dominik A. Ettlin, Nenad Lukic, Marcus Cheetham, Lutz Jäncke, Kai Lutz

**Affiliations:** ^1^Center of Dental Medicine, Clinic for Removable Prosthodontics, Masticatory Disorders and Special Care Dentistry, University of ZurichZurich, Switzerland; ^2^Chiropractic Medicine, Balgrist University HospitalZurich, Switzerland; ^3^MRI Technology, Institute for Biomedical Engineering, Swiss Federal Institute of Technology and the University of ZurichZurich, Switzerland; ^4^Institute of Psychology, Department of Neuropsychology, University of ZurichZurich, Switzerland; ^5^Center for Neurology and Rehabilitation CereneoVitznau, Switzerland

**Keywords:** pain, fear conditioning, tooth, skin conductance response, fMRI, amygdala, medial prefrontal cortex (mPFC), dental phobia

## Abstract

Experimental fear conditioning in humans is widely used as a model to investigate the neural basis of fear learning and to unravel the pathogenesis of anxiety disorders. It has been observed that fear conditioning depends on stimulus salience and subject vulnerability to fear. It is further known that the prevalence of dental-related fear and phobia is exceedingly high in the population. Dental phobia is unique as no other body part is associated with a specific phobia. Therefore, we hypothesized that painful dental stimuli exhibit an enhanced susceptibility to fear conditioning when comparing to equal perceived stimuli applied to other body sites. Differential susceptibility to pain-related fear was investigated by analyzing responses to an unconditioned stimulus (UCS) applied to the right maxillary canine (UCS-c) vs. the right tibia (UCS-t). For fear conditioning, UCS-c and USC-t consisted of painful electric stimuli, carefully matched at both application sites for equal intensity and quality perception. UCSs were paired to simple geometrical forms which served as conditioned stimuli (CS+). Unpaired CS+ were presented for eliciting and analyzing conditioned fear responses. Outcome parameter were (1) skin conductance changes and (2) time-dependent brain activity (BOLD responses) in fear-related brain regions such as the amygdala, anterior cingulate cortex, insula, thalamus, orbitofrontal cortex, and medial prefrontal cortex. A preferential susceptibility of dental pain to fear conditioning was observed, reflected by heightened skin conductance responses and enhanced time-dependent brain activity (BOLD responses) in the fear network. For the first time, this study demonstrates fear-related neurobiological mechanisms that point toward a superior conditionability of tooth pain. Beside traumatic dental experiences our results offer novel evidence that might explain the high prevalence of dental-related fears in the population.

## Introduction

Experimental fear conditioning has proven to be a valuable tool for studying the neurobiological underpinnings of (pain-related) fear, anxiety, specific phobias, and placebo analgesia. (Cheng et al., 2003; Phelps et al., 2004; Delgado et al., 2006; Bradley et al., 2008; Schiller et al., 2008; Lui et al., 2010; De Peuter et al., 2011; Schweckendiek et al., 2011; Dunsmoor et al., 2013). Fear conditioning entails a learning process in which a predictive association is acquired between a previously neutral stimulus (i.e., the conditioned stimulus, CS) and a fear-evoking stimulus (i.e., the unconditioned stimulus, UCS). Following a number of paired presentations of the CS and UCS, the sole presentation of the conditioned stimulus (CS+) is sufficient to elicit an emotional response (conditioned response, CR) similar to that evoked by the UCS.

Regarding the neural basis of fear conditioning, studies point to the amygdala as a key structure of fear learning (Buchel et al., 1998; LaBar et al., 1998; Phelps et al., 2004). But such findings are not consistent. Some studies failed to detect amygdala responses during fear conditioning (Knight et al., 1999, 2004; Fischer et al., 2000, 2002; Jensen et al., 2003). Importantly, a constellation of other structures such as the orbital frontal cortex (OFC), the thalamus, anterior cingulate cortex (ACC), the insula and the medial prefrontal cortex (mPFC) are linked to aspects of fear conditioning (Davis and Whalen, 2001; Phelps et al., 2004; Sehlmeyer et al., 2009; Guhn et al., 2014). These structures modulate fear responses and extend them to the wider context of the conditioning (Fiddick, 2011).

It has been observed that fear conditioning depends on stimulus salience. Interestingly, some classes of stimuli appear to be more readily associated with the UCS, leading to more pronounced CR development and greater resistance to CR extinction. This has been observed for biologically salient stimuli like spiders and angry faces (Ohman and Dimberg, 1978; Ohman and Soares, 1993; Schweckendiek et al., 2011). In support of this observation, Seligman (1971) found that human fears and phobias are not randomly distributed in the population, thus suggesting the presence of specific underlying mechanisms for fear development. Dental phobia is of particular interest in this regard as it is one of the most prevalent phobias and should be considered as a specific phobia (van Houtem et al., 2013). It is a remarkably severe condition with protracted duration and resistancy to treatment (Agras et al., 1969; Fiset et al., 1989; Ost, 1989, 1997; Oosterink et al., 2009). Dental phobia is defined as the excessive and uncontrollable fear of dental treatment, whereas the majority of phobics indicate that fear of pain and feelings of helplessness are the main reasons for their intense dental anxiety (Scharmuller et al., 2014). Furthermore, dental phobia is unique as no other body part is associated with a specific phobia.

It follows from the pertinent literature and the foregoing considerations that dental pain might exhibit enhanced fear responses compared with other bodily pains. Working on this basis that tooth pain is more susceptible to fear conditioning, we expected to find a stronger CR of dental stimuli (CS+c) compared with tibial stimuli (CS+t), the latter serving as a control. After equalizing the UCS pain intensity and quality at both stimulation sites (UCS-c, UCS-t, respectively), we expected differential CRs by analyzing skin conductance responses (SCR) and brain activity (blood oxygenation level dependent, BOLD) in fear-related brain regions (ACC, amygdala, insula, thalamus, OFC, and mPFC).

## Materials and methods

### Subjects

On the basis of a stringent selection process, 21 healthy subjects (mean age = 32.3, *SD* ± 8.2, 12 females) reporting regular visits to dentists (and/or dental hygienists) participated in the study. Exclusion criteria included systemic disease, caries, large restorations, periodontal disease, dental anxiety/phobia or a history of trauma or sensitivity of maxillary canines. Four subjects did not fulfill the criteria of the pain matching procedure (see below for criteria), three subjects were excluded from the SCR analysis due to technical failure of the recording system, and two subjects were excluded because they did not develop contingency awareness. These exclusions resulted in a total sample of *n* = 15 for fMRI analysis and *n* = 12 SCR datasets. The study and all procedures and consent forms were approved by the local Ethics Committee. Subjects received 50 Swiss Francs per hour for participation.

### Interview and anxiety scales

In order to compare the relevance of both stimulation sites for fear, subjects were carefully selected to ensure no history whatsoever of dental or tibial-related anxiety. In an interview session preceding the conditioning experiment and without giving any indication as to the reason for the interview, subjects were required to report experience in any form of a traumatic event at the dentist or dental hygienist or of any injuries to the dentition or tibial region. Potential subjects were excluded from participation if they reported any traumatic event or injury. To exclude possible anxiety-mediated effects associated with dental stimulation, participants completed the Dental Anxiety Scale (DAS), which is one of the most often used dental fear instruments (Corah, 1969). DAS scores below 13 points indicate mild to no dental anxiety. Subjects scoring in excess of 13 points were excluded from further participation. Given the relationship between dental anxiety and general fears and anxiety (Fuentes et al., 2009), we applied also the State-Trait Anxiety Inventory (STAI), the most widely used self-report measure of anxiety (Spielberger et al., 1983). The STAI state is suitable as a screening instrument for predicting anxiety disorders (Kvaal et al., 2005). A cut-off point of 39–40 indicates clinically significant symptoms of state anxiety (Knight et al., 1983). Subjects with a score above 39 points were excluded.

### Electric stimulus delivery

A modified “Compex Motion” system (Compex Médical SA, Ecublens, Switzerland) was used as described by Keller et al. (2002). This stimulation has been proven to evoke reliable sharp and pricking pain sensations (Keller et al., 2002; Brugger et al., 2011, 2012). The Presentation® software (http://www.neurobs.com/presentation) was used to control the experimental protocol. Shielded wires were used to avoid radiofrequency contamination by the stimulation current.

### Tibial stimulus application

**Figure 1:**
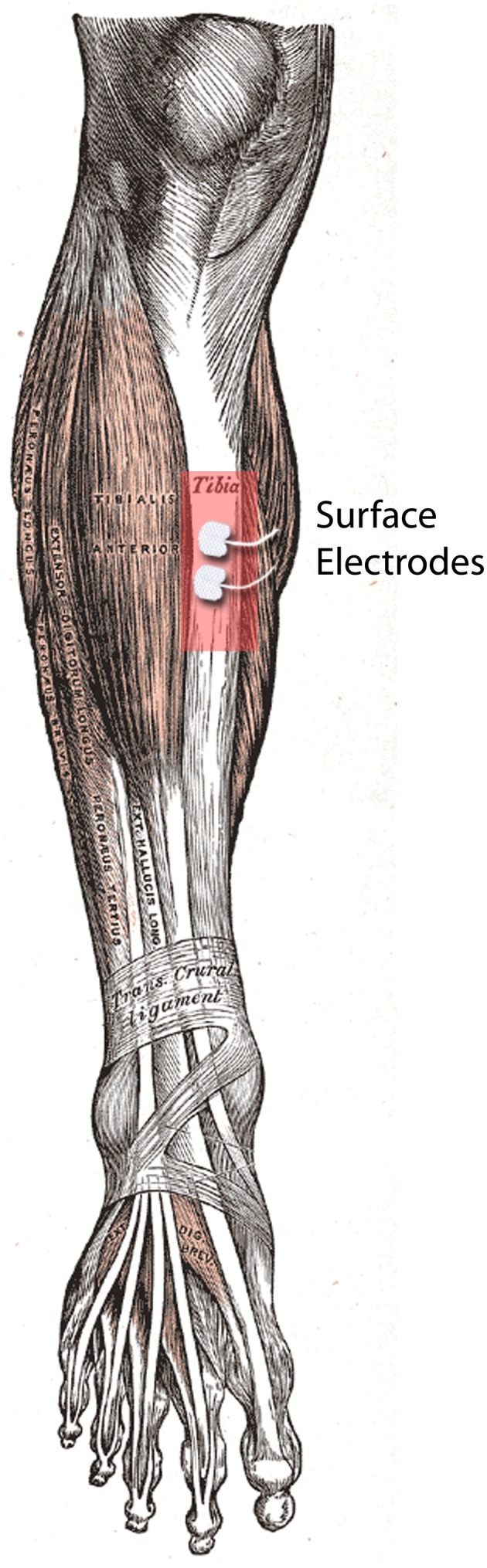
**UCS delivery site (right tibia).** It shows the placement of the electrodes on the anterior border of the tibia.

Small hydrogel surface electrodes (28 × 20 mm, Ambu A/S, Denmark) were used for tibial stimulations (Figure 1). The electrodes were placed on the anterior border of the tibia at a distance of 1 cm. Care was taken that the tibialis anterior muscle was unaffected by the stimulation.

### Dental stimulus application

**Figure 2:**
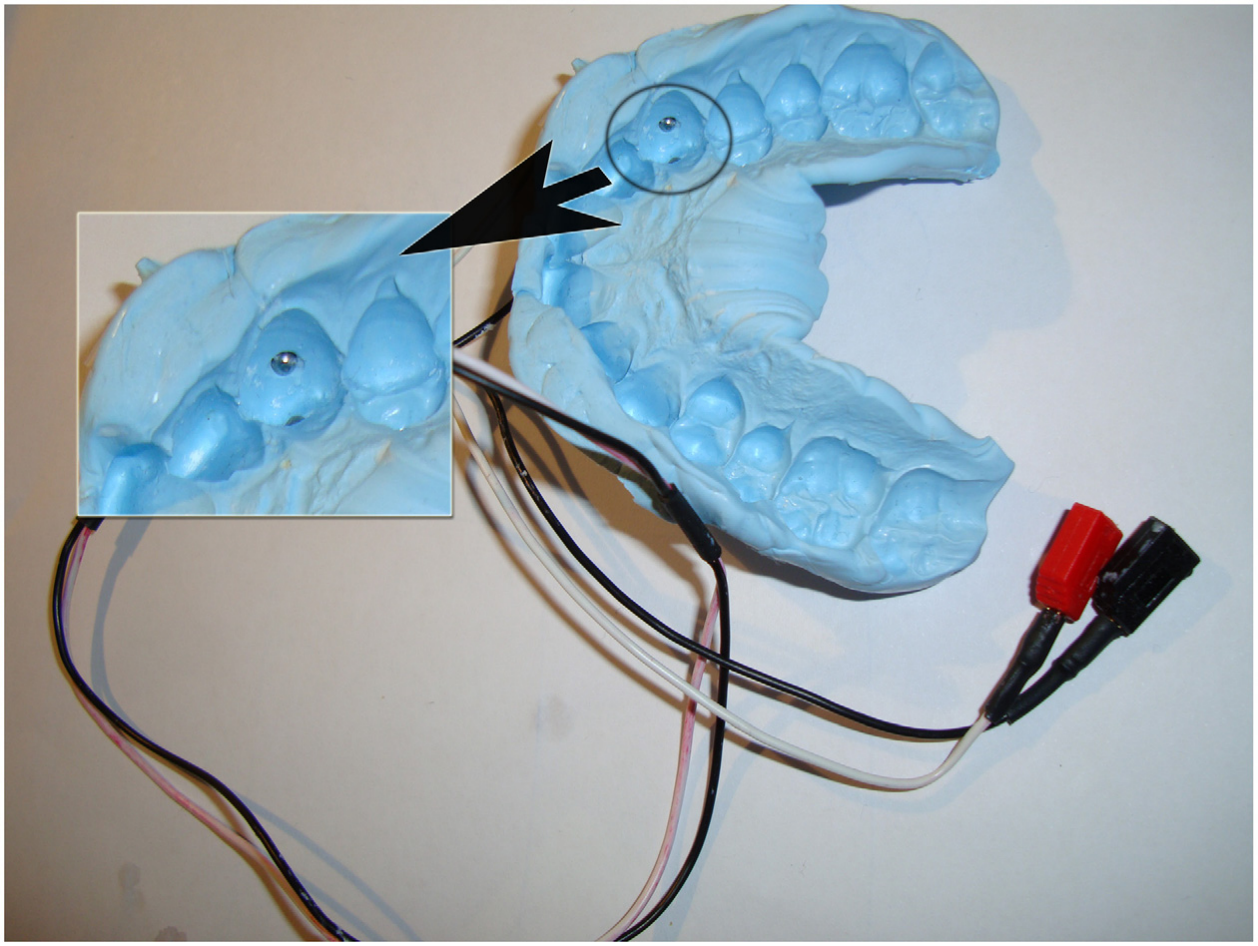
**UCS delivery site (right canine).** This figure illustrates an individual dental splint with embedded electrodes.

Blu-Mousse (Thixotropic Vinyl Polysiloxane, Edgewood, MD, USA) impressions were taken from the subject's dentition (Gutzeit et al., 2011; Meier et al., 2012). Stainless steel electrodes were embedded in each splint at the labial and palatal centers of the right upper canine (Figure 2). To minimize electric resistance, we placed a 3-mm round piece of hydrogel (Klusapotheke, Zurich, Switzerland) on the electrodes. Care was taken that the splint itself did not evoke pain or discomfort.

### Matching of UCS pain intensity and quality

Fiber specificity plays an important role in experimental pain. A-delta and C fibers are major pain-conducting nerve fibers and are thought to activate different cortical regions within the “pain matrix” (Matre et al., 2010). A-delta fibers evoke an initial sharp, pricking, and well-localized pain experience, whereas C fibers elicit dull and prolonged perceptions (Bishop et al., 1958). We aimed to evoke a pricking pain experience at both stimulation sites, thus activating mainly A-delta pain fibers in the following three-step procedure.

Firstly, we applied different intensities of electric current in ascending order and asked subjects to report their respective pain experience as either “pricking,” “dull,” or “pressing.” These three verbal descriptors best permit discrimination between A-delta and C-fiber mediated pain experience with a specificity and sensitivity over 95% (Beissner et al., 2010). Subjects who did not report the perception of pain to be “pricking” were excluded from the study.

**Figure 3:**
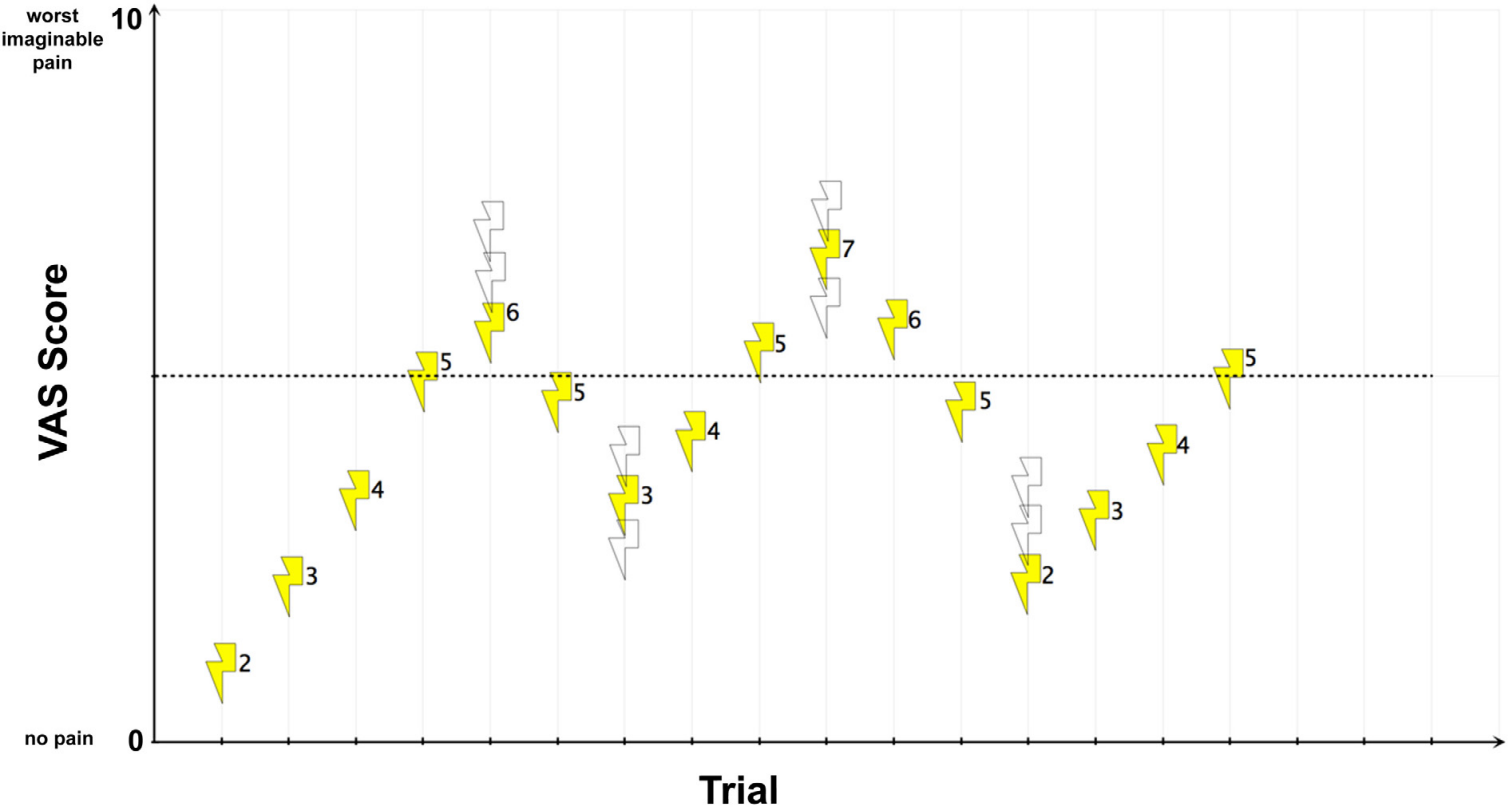
**UCS pain intensity and quality matching.** For illustration, exemplary electric current strengths (mA-values) are shown next to each stimulus. In this example, an electric current strength of 5 mA reached the transition point. Open symbols represent other possible stimulus intensities that might have been chosen by the randomization procedure.

Second, we applied different intensities of electric current according to an adaptive staircase method (Figure 3). This method entails the presentation of a sequence of stimuli, each of which is judged after presentation concerning perceived intensity. The stimulus strength is adjusted to progressively increase or decrease until the judged intensity changes. Upon change, the stimulus intensity is reversed. This technique is widely accepted as robust in the detection of pain thresholds and shows reduced between-session variability and improved reliability compared with other methods (Cornsweet, 1962; Yarnitsky and Sprecher, 1994). In the MR scanner but preceding the conditioning paradigm, subjects were asked to rate the perceived intensity of pain on a visual analog scale (VAS), with the endpoints “0” (no pain) and “10” (worst imaginable pain). Alternating the stimulation site, we applied pulses of electric current in steps of 1 mA with an inter-trial interval randomized between 8 and 12 s. Whenever the rating on a stimulation site exceeded or fell below the hypothetical threshold of “5” (i.e., the transition point corresponding to a painful but tolerable experience), the stimulation algorithm randomly chose for the following stimulation of that particular stimulation site one of the three possible next higher intensities. If, for the following stimulation, the subject rated again a “5” or higher, the stimulus intensity was reversed until the subject rated below a “5.” After this, a random stimulus intensity from one of the three next-lower intensities was applied. If the subject then rated below a “5,” the algorithm reversed again and intensities were increased until the subject rated a “5” or higher. This procedure was performed until stimulation at both stimulation sites reached the transition point four times in succession after alternating between the stimulus sites.

Finally, the intensity of the electric shock was taken as the mean value of the four transition points, serving as the individual UCS for each stimulation site. Subjects who did not reach the transition point of “5” within each of the four runs were excluded. To guarantee stable perceptions of stimulus intensity, the whole pain matching procedure was repeated after the extinction phase. To allow for parametric testing of the UCS ratings, we performed a Kolmogorov-Smirnov test which tested for normality of the data. To further control for differences in perceived pain quality, post-experiment valence ratings (unpleasant/pleasant) were collected by using a five-point self-assessment manikin (SAM) scale. To assess possible differences in mean ranks, the non-parametric Wilcoxon signed-rank test was used. Furthermore, subjects who reported a difference in UCS valence of more than one point were excluded.

### Fear conditioning procedure

The experiment consisted of an acquisition phase followed by an extinction phase (30 unreinforced trials, 10 per CS). Only data of the acquisition phase is reported in the present study. During the acquisition phase a 50% partial reinforcement conditioning strategy was applied which allowed for a UCS-free comparison of both CS. This approach was successfully used in other fear conditioning experiments and permits the analysis of fear responses without confounding effects of the UCS (Buchel et al., 1998; Dunsmoor et al., 2007; Moessnang et al., 2013). The three CS consisted of simple geometrical forms: a triangle, a circle, and a square. These were presented in a pseudo-randomized order (no more than two consecutive trials) and in white color on a black background (CS duration 2 s, inter-trial interval 8–12 s). Assignment of the geometrical form to the different US was randomized across subjects. One CS (CS−) was never paired with an electric shock. The UCS, having a duration of 1 ms, co-terminated with the CS presentation. During the acquisition phase, a total of 150 visual stimuli were presented. These consisted of 30 CS−, 30 unconditioned and conditioned stimuli of each type (UCS-c, UCS-t, and CS+c, CS+t, respectively). Subjects were instructed that each of the geometrical forms could be followed by an electric shock, either to the canine tooth or to the shinbone.

#### Contingency awareness

Although still debated, controlling for contingency awareness is important in order to reduce differences in the dependent variables (Lovibond and Shanks, 2002; Hamm and Weike, 2005; Tabbert et al., 2011). Subject awareness of the reinforcement contingencies was assessed immediately after the extinction phase in an interview conducted in the control room outside the magnet. Subjects were asked to choose which type of geometric figure preceded the different UCS types using a forced choice questionnaire.

### Skin conductance responses

SCR were acquired using the constant voltage (0.5 V) method by means of MRI-compatible and radiotranslucent electrodes with a 1 cm diameter contact area placed on the distal phalanges of the second and third finger of the participant's left hand (BIOPAC Systems Inc., Goleta, CA). The SCR signal was amplified and recorded with a BIOPAC Systems skin conductance module connected to an Apple MacBook Pro running AcqKnowledge software version 4.0 (BIOPAC Systems Inc., Goleta, CA). Data were recorded with a sampling rate of 200 Hz. The RF-artifacts in the SCR-waveforms were removed off-line by a median-filter (window length: 50 samples) using the software MATLAB R2011b (MathWorks, Natick, MA). Off-line analysis of SCR waveforms was done using the automated scoring system for EDA data included in the AcqKnowledge software. The window length was set to 6 s, starting at the CS presentation. Only SCRs were analyzed with response amplitude higher than 10% of the maximal response. The SCRs were then normalized through a square root transformation. Statistical analyses were performed using paired *t*-tests as implemented in the software PASW Statistics (Version 18, SPSS Inc.). To be consistent with the fMRI analysis (see below), we divided the acquisition phase in an early (3rd to 16th trial) and late phase (17th to 30th trial).

### fMRI protocol

Functional and anatomical scans were obtained using a 3-T Phillips Achieva scanner with an 8-channel receive-only head coil. We used a blood-oxygen-level dependent (BOLD) sensitive single-shot gradient echo-planar imaging sequence to acquire 33 axial whole brain slices. Parameters were as follows: echo time = 30 ms, flip angle = 75°, repetition time = 2526 ms, slice thickness = 4 mm, inter-slice gap = 0 mm, field of view = 220 mm, and matrix size in plane = 128 × 128, resulting in a voxel size of 1.72 × 1.72 × 4 mm^3^. Three dummy scans were first acquired to reach steady-state magnetization and subsequently discarded. 180 high-resolution T1- weighted axial slices (spoiled gradient echo) were acquired with *TR* = 20 ms, flip angle = 20°, voxel size = 0.98 × 0.98 × 1.02 mm^3^, FOV = 24 cm, and matrix = 256 × 192; these were used as an underlay for individual functional maps. The acquisition phase of 930 functional images lasted about 28 min and was followed by an extinction phase of ~10 min.

SPM8 (http://www.fil.ion.ucl.ac.uk/spm) software package running on MATLAB R2011b (Mathworks, Natiek, USA) was used for functional voxel-by-voxel analysis. After slice timing, spatial realignment to the first image in the series as reference was performed and it was assured that detected movement did not exceed 2 mm (translational) or 1° (rotational) in relation to the reference. For studying group effects, data were normalized to the MNI template brain (Evans et al., 1992) followed by smoothing with a Gaussian kernel of 8 mm full-width-at-half-maximum (FWHM). To control for possible head movement effects, individual movement parameters (translations in x, y, and z-direction, as well as rotations around x, y, and z axis) were implemented in the 1st level model as regressors of no interest. Individual SCR amplitudes (*N* = 12) were included as additional regressors of no interest to account for possible differences in brain activity explained by differential SCR levels. The first two trials of each CS were discarded from analysis because learning could not have occurred yet (Phelps et al., 2004; Schweckendiek et al., 2011; Merz et al., 2013). The high-pass filter was set to 128 s and the regressors were convolved with the canonical hemodynamic response function implemented in SPM8. To account for gradual development of fear expression, we divided the acquisition phase in an early (3rd to 16th trial) and late phase (17th to 30th trial) (LaBar et al., 1998; Tabbert et al., 2005; Schiller et al., 2008). For each subject, the following experimental conditions were modeled: CS+c, CS+t, CS− (early and late phase each), UCS-c and UCS-t. The CS regressor onsets were set to coincide with the presentation of the CS with a duration of 2 s. The UCS onsets were set 2 s after CS presentation. Statistical parametric maps were then calculated, yielding beta estimates of the model fit for each subject and condition. The random effects group analysis was performed by using one-sample *t*-tests. The contrasts CS+c > CS−, CS+t > CS−, CS+c > CS+t, and CS+t > CS+c were computed for the early and late phase of the acquisition. Resulting voxel *T*-values were color-coded and superimposed onto the MNI single-subject-T1 brain using MRIcroGL (http://www.cabiatl.com/mricrogl/). For visualization purposes, we used a whole-brain statistical threshold of *p* < 0.001 (uncorrected) with a voxel extend threshold of 10 voxels.

In a subsequent region-of-interest (ROI) analysis, we investigated the following bilateral brain structures: amygdala, insula, ACC, OFC, thalamus, and the mPFC. The ROI masks were taken from the probabilistic Harvard-Oxford Cortical and Subcortical Structural Atlas (http://www.fmrib.ox.ac.uk/fsl). The probability threshold for belonging to the respective brain region was set to *p* > 0.25. To further assess the success of the UCS matching procedure, we additionally introduced the posterior part of the insula as a control region which was parcellated after Brooks (Brooks et al., 2002, 2005). This part of the insula is related to sensory aspects of pain (Craig, 2009; Garcia-Larrea, 2012) and appears to be the only part of the cerebral cortex where intra-cortical electric stimulation is able to trigger experience of somatic pain (Mazzola et al., 2012). The failure to find significant differences between UCS-c and UCS-t in this region would provide additional support for the equivalence of subjective pain intensities. All ROI analyses were computed using the small volume correction implemented in SPM8. Only clusters which survived a familywise error rate (FWE) correction were reported.

## Results

### Anxiety scales and interview

None of the subjects recalled any traumatic event at the dentist or dental hygienist or any traumatic injuries in the tibia region. All subjects showed scores for state and dental anxiety in a low, non-clinical range, with a mean DAS score of 7.46 (*SD* ± 1.50) and a mean STAI score of 29.35 (*SD* ± 4.51).

### Pain intensity and quality matching

**Figure 4:**
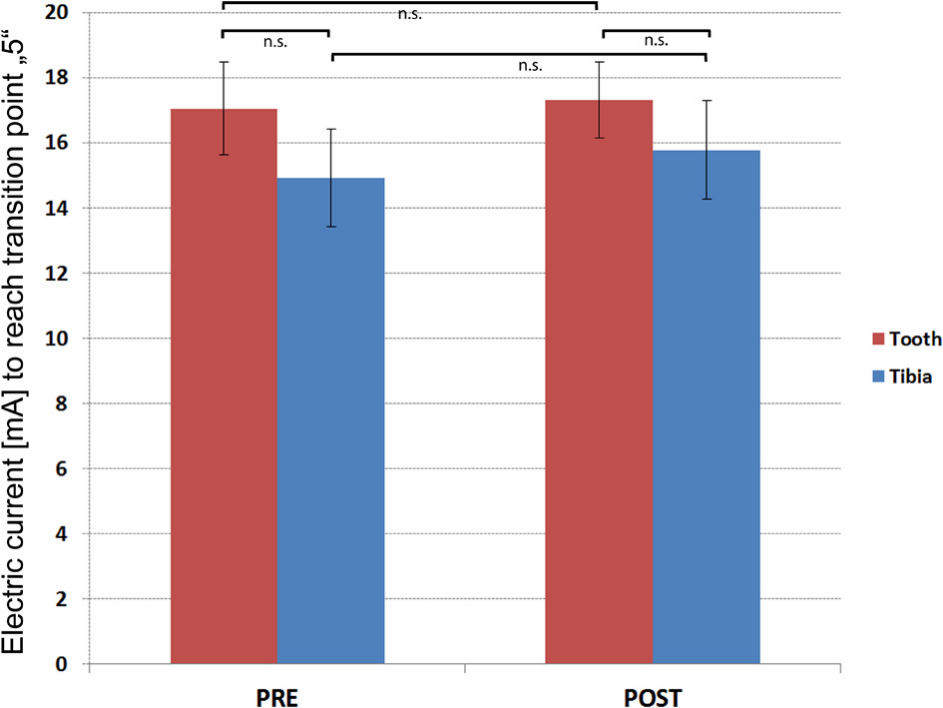
**Results of the pain matching.** Y-axis illustrates the group (*N* = 15) mean electric current (mA) that was needed to reach the transition point. T-bars indicate standard errors of the mean (±s.e.m.).

None of the participants reported any painful or uncomfortable sensations associated with the dental splint or tibial electrodes themselves. Four of twenty-one participants did not reach the transition point of “5” on the VAS scale and were excluded. The Kolmogorov-Smirnov test indicated normality of the UCS rating data (*Z* = 0.90, *p* = 0.39). Pre- and post-experiment pain matching revealed that slightly higher currents were needed for the canine tooth to reach the transition point compared with tibial stimulations (pre-experiment mA-values [Mean ± s.e.m.]; Tooth: 17.04 ± 1.42, Tibia: 14.90 ± 1.49/post-experiment mean mA-values [Mean ± s.e.m.]; Tooth: 17.32 ± 1.17, Tibia 15.77 ± 1.52) (Figure 4). However, these differences were not significant (pre-experiment *T* = 0.74; *p* = 0.48/post-experimental *T* = 0.43; *p* = 0.68). Furthermore, pre- and post-experiment differences within UCS-c and UCS-t intensities were not significant (UCS-c: *T* = −0.16; *p* = 0.88/UCS-t: *T* = −0.40; *p* = 0.70). To control for sensitization or habituation effects, or any other changes in perception of the electric stimulus, we calculated the intraclass correlation coefficient (ICC) between individual pre-and post-experiment electric current strengths required during the pain matching procedure to reach the transition point. We observed an ICC of 0.788 (*F* = 8.419, *p* = 0.001) for UCS-t- and 0.745 (*F* = 6.857, *p* = 0.003) for UCS-c-intensities, pointing toward highly stable thresholds. Regarding pain quality, all participants reported a short and pricking pain perception. Furthermore, post-experimental SAM ratings did not show any significant differences between UCS-c and UCS-t, as revealed by Wilcoxons test (*Z* = −1.385; *p* = 0.166).

### Skin conductance responses

#### Early phase of acquisition

**Figure 5:**
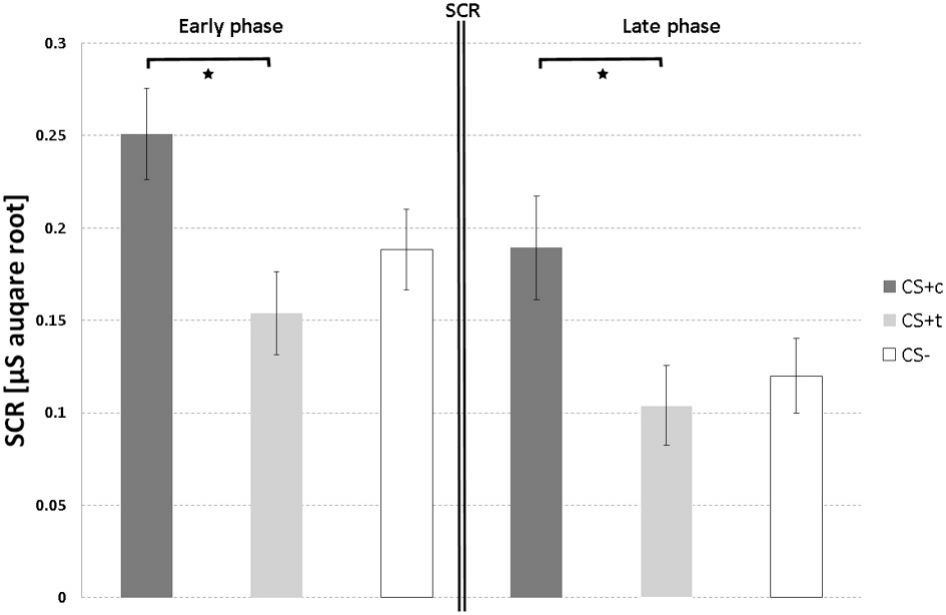
**SCR responses (μS, square root transformed, *N* = 12) over both acquisition phases.** T-bars represent standard errors of the mean (±s.e.m.). ^*^p < 0.05.

Paired *t*-tests of the autonomic responses of CS+c revealed significantly stronger SCR compared to CS+t (*T* = 2.28, *p* = 0.02), although both stimuli were rated as equally painful (Figure 5). No significant differences could be found between CS+c and CS− and CS+t and CS−.

#### Late phase of acquisition

As in the early phase, paired *t*-tests of CS+c showed significantly stronger SCR than the CS+t (*T* = 2.39, *p* = 0.02) (Figure 5). Again, no significant differences could be found between both CS+ and CS−.

### fMRI results

#### Unconditioned responses

**Figure 6:**
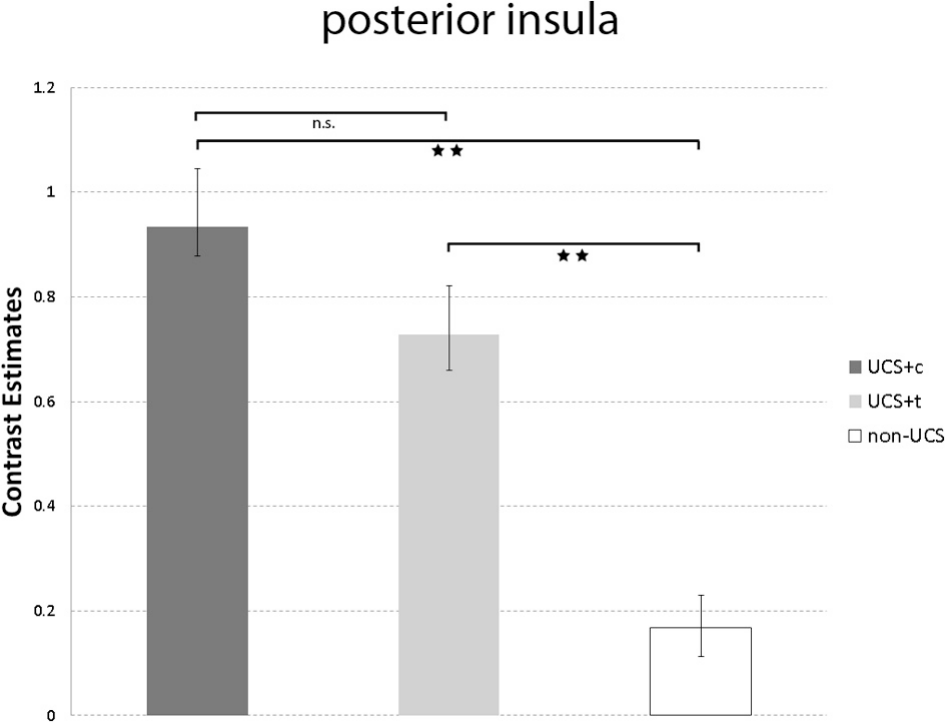
**Illustrated are the mean contrast estimates of the unconditioned responses UCS-c, UCS-t, and Non-UCS in the posterior insula.** T-bars represent standard errors of the mean (±s.e.m.). ^**^p < 0.01.

Figure 6 shows the comparison between UCS-c and UCS-t in the posterior insula ([Mean contrast estimates ± s.e.m.]; UCS-c: 0.93 ± 0.21. UCS-t: 0.73 ± 0.15). The paired *t*-tests revealed no significant results (*T* = 0.68, *p* = 0.51). However, both UCS showed significantly higher activation compared to the non-UCS (UCS-c vs. non-UCS: *T* = 2.95, *p* = 0.01; UCS-t vs. non-UCS: *T* = 3.65, *p* = 0.01).

#### Conditioned responses

For the early and late phase of acquisition, peak coordinates, *t*-values and corrected *p*-values of the respective contrasts are shown in Table 1.

**Table 1:** **Results of the conditioned responses in early and late phases of the acquisition phase**.

**Acquisition phase**	**Contrast**	**Brain region**	**T_max_**	**p_corr_**	***x***	***y***	***z***
Early phase	CS+c > CS−	Left aMCC	4.31	0.014	−2	22	36
		Right aMCC	4.30	0.014	6	32	26
		Right amygdala	3.80	0.022	30	−6	−22
		Left anterior insula	4.78	0.026	−30	22	2
		Right anterior insula	5.40	0.001	36	16	−14
		Left OFC	5.13	0.001	−30	24	−8
		Right OFC	5.45	0.001	34	22	−20
		Left thalamus	3.59	0.038	−12	−12	0
	CS+t > CS−	No significant results					
	CS+c > CS+t	Left aMCC	5.66	0.001	−4	28	32
		Right aMCC	5.51	0.001	0	26	34
		Left anterior insula	4.96	0.001	−34	20	2
		Right anterior insula	5.19	0.001	38	14	−8
		Left OFC	4.63	0.006	−36	26	0
		Right OFC	4.82	0.003	36	20	−22
		Left thalamus	4.34	0.011	−10	−8	4
		Right thalamus	4.13	0.022	8	−8	4
	CS+t > CS+c	No significant result					
Late phase	CS+c > CS−	No significant results					
	CS+t > CS−	No significant results					
	CS+c > CS+t	No significant results					
	CS+t > CS+c	Right mPFC	3.41	0.042	4	52	−4

Peak voxels and p-values are shown for the contrasts CS+c > CS−, CS+t > CS−, CS+c > CS+t, and CS+t > CS+c. The threshold for the ROI analysis (small volume correction) was set to p < 0.05 (FWE-corrected according to SPM8). Coordinates are reported in the MNI space.

#### Early phase of acquisition

**Figure 7:**
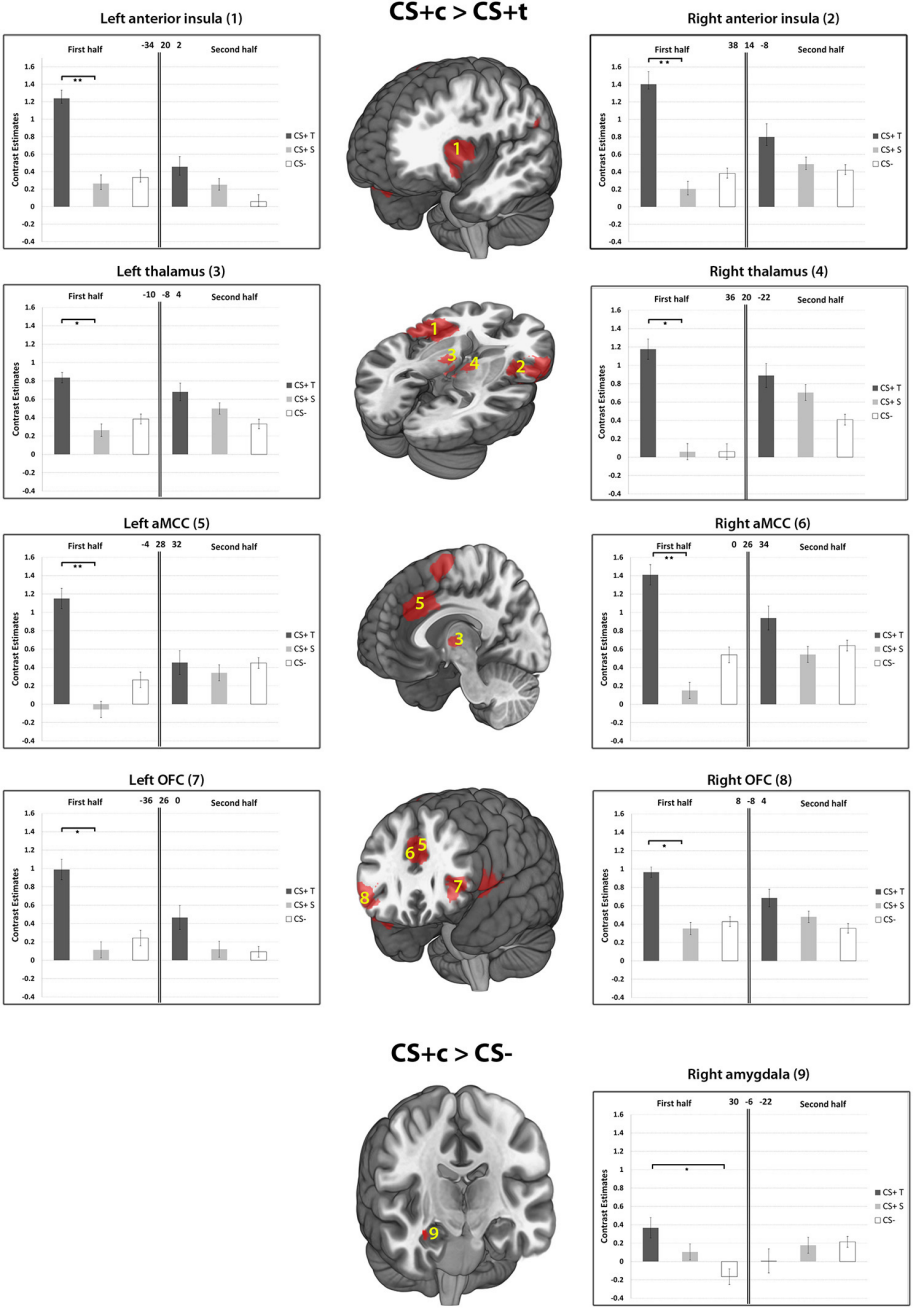
**Results of the contrasts CS+c > CS+t, and CS+c > CS− within ROIs 1–9.** Whole-brain SPM activations maps are shown with a statistical threshold of *p* < 0.001, uncorrected, voxel threshold = 10. Mean contrast estimates (and standard errors of the mean ± s.e.m.) for early and late phases in the respective peak voxels are illustrated in the bar graph. ^*^p < 0.05; ^**^p < 0.01.

The whole-brain analysis of the contrast CS+c > CS- revealed a single cluster in the right OFC (Peak MNI 34 22 −20, *T* = 5.45, *p* < 0.05, FWE-corrected). The respective ROI analysis (based on small-volume correction) revealed significantly higher responses in the bilateral anterior midcingulate cortex (aMCC), the right amygdala, bilaterally in the anterior insula, the OFC and thalamus (*p* < 0.05, FWE-corrected). Regarding the contrast CS+t > CS- no significant activations could be found. The comparison CS+c > CS+t revealed a significant cluster in the left aMCC in the whole-brain-analysis (Peak MNI −4 28 32, *T* = 5.66, *p* < 0.05, FWE-corrected). Further ROI analysis yielded significant activations bilaterally in the anterior insula, OFC, and Thalamus (Figure 7). The reverse contrast CS+t > CS+c did not show any significant results.

**Figure 8:**
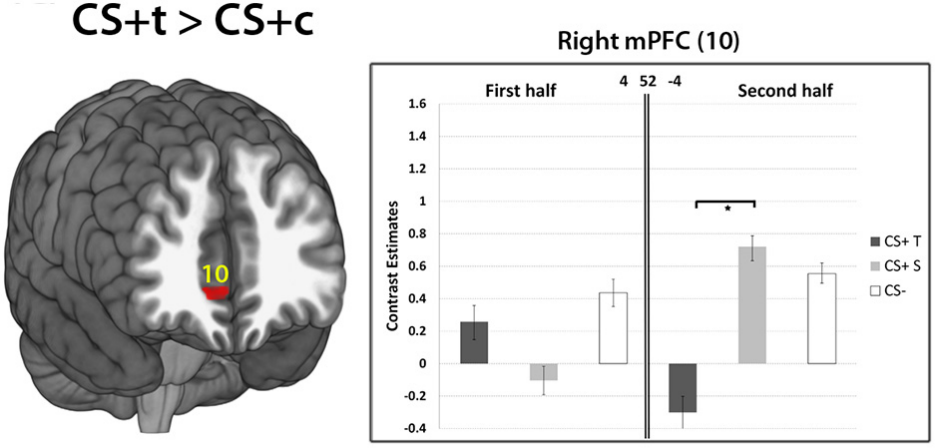
**Results of the contrast CS+t> CS+c within the mPFC ROI (10).** Whole-brain SPM activations maps are shown with a statistical threshold of *p* < 0.001, uncorrected, voxel threshold = 10. Mean contrast estimates (and standard errors of the mean ± s.e.m.) for early and late phases in the respective peak voxels are illustrated in the bar graph. ^*^p < 0.05.

***Late phase of acquisition*.** The contrast CS+c > CS- revealed no significant results. Similary, the comparisons CS+t > CS− and CS+c > CS+t did not show any significant results. However, the contrast CS+t > CS+c showed significantly higher responses in the mPFC ROI (*p* < 0.05, FWE-corrected, Figure 8).

## Discussion

In the current study, we asked the question whether painful stimuli applied at the tooth and tibia evoke different fear responses while having subjectively identical intensity and quality. The finding of such selectivity in fear responses of healthy subjects would lend weight to the idea that the underlying brain mechanisms responsive to the two different sites are not quite the same and that this difference is potentially associated and thus contributes in some way to the development of specific phobias such as dental phobia. In order to directly compare brain activity and SCR between anticipated dental and tibial shocks, it was crucial to match the UCS at both stimulation sites in subjectively perceived pain intensity and quality. The success of our UCS matching procedure is not only depicted in pre- and post-experiment measurements, but also in non-significant differences between UCS-c and UCS-t responses in the posterior insula. This part of the insula has been proposed as a potential “primary cortex for pain” (Garcia-Larrea, 2012) and constitutes a promising biomarker for pain (Wager et al., 2013).

As hypothesized, our results provide strong evidence in favor of heightened susceptibility of CS+c to fear conditioning in subjects without a history of dental fear. This evidence is provided on the basis of two independent but concurrently applied methods, namely SCR and BOLD responses. As a main finding, enhanced brain activation of CS+c compared to CS+t could be found in regions of the fear network including the aMCC, the anterior insula, the OFC and the thalamus. These activations were exclusively present in the early phase of acquisition, which is in line with other studies reporting fear related brain activation in the first half of the acquisition phase (Schiller et al., 2008; Schweckendiek et al., 2011). Enhanced responses of the amygdala could only be found in the comparison CS+c > CS−. Several lines of evidence point toward the amygdala as a key neural system underpinning fear learning and extinction (LeDoux, 1996; Buchel et al., 1998; LaBar et al., 1998). However, a recent review of 44 fear conditioning studies showed that 19 of these failed to find amygdala activation (Sehlmeyer et al., 2009). Previous results from fear conditioning studies indicate that the amygdala is involved during the initial learning phase only, showing rapid habituation after a few trials (Buchel et al., 1998; LaBar et al., 1998; Marschner et al., 2008; Bach et al., 2011). The OFC has also been implicated in aspects of fear learning and has been labeled the “extended amygdala,” together with other structures such as the bed nucleus of the stria terminalis (Davis and Whalen, 2001; Fiddick, 2011). The finding of enhanced amygdala and OFC activity solely in the first half of the CS+c > CS− condition supports our hypothesis regarding enhanced susceptibility of of CS+c to fear conditioning.

There have long been doubts about the adequacy of animal fear conditioning models (which favor the amygdala as a core structure) in explaining anxiety disorders (Fiddick, 2011). Recently, this traditional view of the amygdala has been extended by an involvement of several other brain regions which play an important role in fear learning and expression. In maintaining extensive inputs from the amygdala (Vogt, 2005), the ACC is involved in the anticipation of threat, aware conditioning, response selection, and in the interpretation of interoceptive states (Paulus and Stein, 2006; Mechias et al., 2010; Merz et al., 2013). These interoceptive states are integrated in the anterior insula (Craig, 2002) and are often associated with intensive aspects of affective components which can provoke strong withdrawal actions. It has been proposed that this neural circuit including the anterior insula and the ACC plays an important role regarding salience (Downar et al., 2003; Iannetti and Mouraux, 2010) and “anxiety sensitivity,” a term which is used to describe the tendency of certain individuals to view interoceptive sensations as dangerous and threatening (Reiss et al., 1986; Paulus and Stein, 2006). Our results of increased responses of CS+c compared to CS+t in the anterior insula and aMCC in healthy subjects point toward enhanced emotional salience and fear relevance of painful dental stimuli although the subjects received an equal aversive UCS at the tibia. Furthermore, the enhanced co-activity of the aMCC and the anterior insula of CS+c might be linked to an increased functional connectivity between these two brain areas that recently has been shown to be associated with heightened threat value of an impending stimulus (Wiech et al., 2010). In conceptualizing the role the anterior insula, the ACC and the amygdala in fear expression, Fiddick (2011) proposes a distinction between fear-provoking immediate (amygdala) and anxiety-provoking potential (anterior insula, ACC) threats. Accordingly, the current results indicate some form of concurrent and increased involvement of both fear-provoking and anxiety provoking systems regarding CS+c.

Interestingly, the contrast CS+t > CS+c revealed significantly greater activations in the mPFC within the late phase of the experiment. Activity in the mPFC has been frequently reported in fear conditioning studies (Phelps et al., 2004; Schiller et al., 2008; Sehlmeyer et al., 2009). Beside emotion regulation, the mPFC is associated with fear extinction which occurs when a CS is presented alone, without the UCS, eventually leading to an elimination of the CR (Morgan et al., 1993; Phelps et al., 2004). Moreover, there is evidence for a strong functional coupling between the mPFC and amygdalar nuclei as the mPFC exerts inhibitory control over the amygdala and therefore inhibits fear responses (Phelps et al., 2004; Schweckendiek et al., 2011). Enhanced activity in the mPFC of CS+t compared to CS+c might point toward less efficient extinction mechanisms of CS+c which supports clinical observations of enhanced resistance of dental phobia to treatment compared to other specific phobias (Ost, 1989, 1997). Since this difference in mPFC activity only appears at the later stage of the conditioning phase in the experiment, this might allow to speculate about a possible re-evaluation of the CS+t during the late phase: its potential to elicit threat might decrease due to the mPFC activity. This mechanism is in line with the findings of a study of Schiller et al. (2008) which showed stronger mPFC activity to a safety stimulus that previously predicted danger.

However, the picture of the comparisons to the safe CS− stimuli is not so clear: while the contrast CS+c > CS− shows enhanced activations in all investigated fear related brain regions including the amygdala, the contrast CS+t > CS− revealed no significant results. The same result is depicted in the SCR analysis where no significant differences could be found between autonomic responses of both CS+ and CS−. Although other fear conditioning studies also failed to find differential SCRs regarding CS+ vs. CS− comparisons (Olatunji, 2006; Klucken et al., 2009; Schweckendiek et al., 2011), our results are in contrast to most fear conditioning studies. However, the current study differs from traditional fear conditioning paradigms which operationalized the CR as the difference between CS+ and CS− by using two CS+ presentations and an equalized UCS for both CS+ within one experimental group. This approach might reveal effects such as superior conditionability of one CS+, while the other CS+ indicates a less threatening stimulus which can't be distinguished from the safe CS− on a neural level. These findings have to be interpreted in terms of the larger literature once the present results have been corroborated in further studies.

Finally, as a limitation of the study, we cannot rule out the effects of spatio-temporal contiguity of dental CS-UCS assocations. The formation of CS-UCS associations may be more effective when spatio-temporal contiguity between the CS and UCS is higher. In the present study the CS was a visual stimulus presented on a computer monitor. The spatial contiguity of such CS with the UCS-c is higher than with the UCS-t, and as a result, may more effectively recruit fear networks in the brain. However, due to the fast nerve conduction velocity of A-delta fibers (max. 30 m/s) this effect, if it exists at all, might be minimal. Furthermore, differential effects of fear might be related to the perception of the covariation between fear-relevant stimuli and shock (Tomarken et al., 1989). As we did not assess contingency awareness as quantified by the probability to get the UCS, we cannot rule out such effects.

To conclude, the current study demonstrates new evidence toward neurobiological mechanisms that might contribute to a superior conditionability of tooth pain. Beside classical conditioning effects at dental offices our results offer a novel approach to explain the high prevalence of dental-related fears in the population.

## Author contributions

All authors have discussed the results and commented on the manuscript. Substantial contributions to conception and design—Michael L. Meier, Nuno M. P. de Matos, Kai Lutz. Lutz Jäncke, Nenad Lukic, Acquisition of data, analysis and interpretation of data: Michael L. Meier, Nuno M. P. de Matos, Marcus Cheetham, Dominik A. Ettlin, Drafting the article or revising it critically for important intellectual content: Michael L. Meier, Nenad Lukic, Marcus Cheetham, Dominik A. Ettlin, Mike Brügger, Lutz Jäncke, Kai Lutz, Final approval of the version to be published: Michael L. Meier, Nenad Lukic, Marcus Cheetham, Kai Lutz, Dominik A. Ettlin, Mike Brügger, Lutz Jäncke.

### Conflict of interest statement

The authors declare that the research was conducted in the absence of any commercial or financial relationships that could be construed as a potential conflict of interest.
